# Differential interactions of bacterial lipopolysaccharides with lipid membranes: implications for TRPA1-mediated chemosensation

**DOI:** 10.1038/s41598-018-30534-2

**Published:** 2018-08-13

**Authors:** Justyna B. Startek, Karel Talavera, Thomas Voets, Yeranddy A. Alpizar

**Affiliations:** Laboratory of Ion Channel Research, Department of Cellular and Molecular Medicine. KU Leuven; VIB Center for Brain & Disease Research, Leuven, Belgium

## Abstract

Bacterial lipopolysaccharides (LPS) activate the TRPA1 cation channels in sensory neurons, leading to acute pain and inflammation in mice and to aversive behaviors in fruit flies. However, the precise mechanisms underlying this effect remain elusive. Here we assessed the hypothesis that TRPA1 is activated by mechanical perturbations induced upon LPS insertion in the plasma membrane. We asked whether the effects of different LPS on TRPA1 relate to their ability to induce mechanical alterations in artificial and cellular membranes. We found that LPS from *E*. *coli*, but not from *S*. *minnesota*, activates TRPA1. We then assessed the effects of these LPS on lipid membranes using dyes whose fluorescence properties change upon alteration of the local lipid environment. *E*. *coli* LPS was more effective than *S*. *minnesota* LPS in shifting Laurdan’s emission spectrum towards lower wavelengths, increasing the fluorescence anisotropy of diphenylhexatriene and reducing the fluorescence intensity of merocyanine 540. These data indicate that *E*. *coli* LPS induces stronger changes in the local lipid environment than *S*. *minnesota* LPS, paralleling its distinct ability to activate TRPA1. Our findings indicate that LPS activate TRPA1 by producing mechanical perturbations in the plasma membrane and suggest that TRPA1-mediated chemosensation may result from primary mechanosensory mechanisms.

## Introduction

Lipopolysaccharides (LPS) are major components of the outer membrane of gram-negative bacteria^1,2^ and are key cues for the detection of infection by the immune system^3^. The classical mechanism of LPS recognition involves stimulation of Toll-like receptor 4 (TLR4)^1,4,5^, but we recently showed that LPS activates the Transient Receptor Potential (TRP) cation channels TRPA1^6^ and TRPV4^7^ in a TLR4-independent manner, as well as other TRP channels in sensory neurons^8^. LPS-induced activation of TRPA1 triggers acute local inflammation and pain in mice^6^ and avoidance during feeding and oviposition in *Drosophila melanogaster*^9^. Activation of TRPV4 in airway epithelial cells leads to protective responses such as nitric oxide production and increased ciliary beat frequency^7^. These findings revealed TRP channels as effectors of LPS, operating in excitable and non-excitable cells.

The LPS structure consists of three parts: a hydrophobic region called lipid A, an oligosaccharide core and an O-polysaccharide chain^10^. The lipid A moiety acts as anchor in the bacterial membrane and is formed by highly conserved di-glucosamine backbone and acyl chains that are the primary determinants of bacterial endotoxicity. The lipid A structure varies across bacterial strains, displaying different acyl chain lengths, number and saturation, and are attached to the oligosaccharide backbone with distinct degrees of symmetry^10^. We showed previously that LPS with conically-shaped lipid A (such as that from *E*. *coli*) activate TRPA1 stronger than those forming symmetrical lamellar structures, such as *S*. *minnesota* LPS^6^. These findings suggest that LPS activate these channels by mechanically perturbing the plasma membrane. Although LPS are known to insert in artificial lipid membranes^11–14^, their effects on natural membranes remain largely unknown. Using fluorescent LPS we confirm that both *E*. *coli* and *S*. *minnesota* LPS intercalate into cellular membranes. To investigate the mechanisms underlying TRPA1 activation, we tested whether the capacity of different LPS to activate this channel relates to their ability to induce mechanical alterations in artificial and cellular membranes. For this we first sought to confirm the distinct action of *E*. *coli* LPS and *S*. *minnesota* LPS on TRPA1 when applied in a wide concentration range (1 to 500 µg/ml). Then we assessed the effects of these LPS on giant unilamellar vesicles (GUV) and mammalian cellular membranes by monitoring the changes in fluorescence properties of three dyes sensitive to the order of the lipid environment. We found that *E*. *coli* LPS was more effective than *S*. *minnesota* LPS in activating TRPA1 and in inducing changes in the arrangement of membrane lipids. These results support the hypothesis that TRPA1 serves as LPS sensor by detecting mechanical alterations in the plasma membrane. Ultimately, this may help explaining the distinct ability of different LPS molecules to induce acute neurogenic inflammation^6^.

## Results

### Differential effects of LPS from *E*. *coli* and *S*. *minnesota* on TRPA1

**Figure 1:**
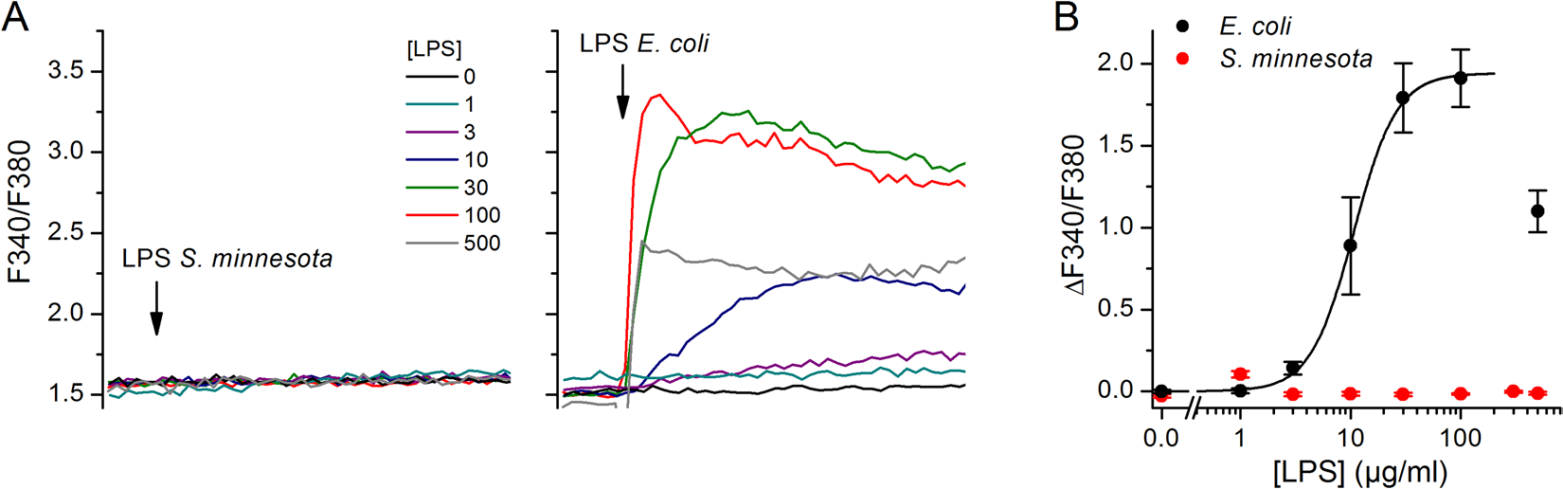
*E*. *coli* LPS, but not *S*. *minnesota* LPS, activates mouse TRPA1. (**A**,**B**) Representative traces of average intracellular Ca^2+^ levels recorded in TRPA1-CHO cells in control and in the presence of LPS from *S*. *minnesota* (left panel) and *E*. *coli* (right panel). The arrows indicate the time point when LPS was applied. (**B**) Concentration dependence of the amplitude of the responses to *S*. *minnesota* and *E*. *coli* LPS. The black line represents the fit of a Hill equation to the *E*. *coli* LPS data (effective concentration = 10.6 ± 0.4 µg/ml and Hill number = 2.2 ± 0.2).

We have previously shown that, at a concentration of 20 µg/ml, LPS extracted from *E*. *coli*, but not from *S*. *minnesota*, activates TRPA1^6^. In intracellular Ca^2+^ imaging experiments performed in CHO cells stably transfected with mouse TRPA1 we now confirm that *E*. *coli* LPS activates TRPA1, whereas *S*. *minnesota* LPS is inactive at concentrations up to 500 µg/ml (Fig. 1).

The amplitude of the response of cells to 500 µg/ml *E*. *coli* LPS was smaller than for 30 and 100 µg/ml. This may be due to a bimodal effect of LPS on TRPA1, whereby this compound acts as agonist at lower concentrations and as antagonists at higher concentrations. This behavior may be reminiscent of that of many other TRPA1 chemical modulators, such as menthol, nicotine, allyl isothiocyanate and cinnamaldehyde^15–18^.

### Imaging the insertion of LPS into cellular membranes

**Figure 2:**
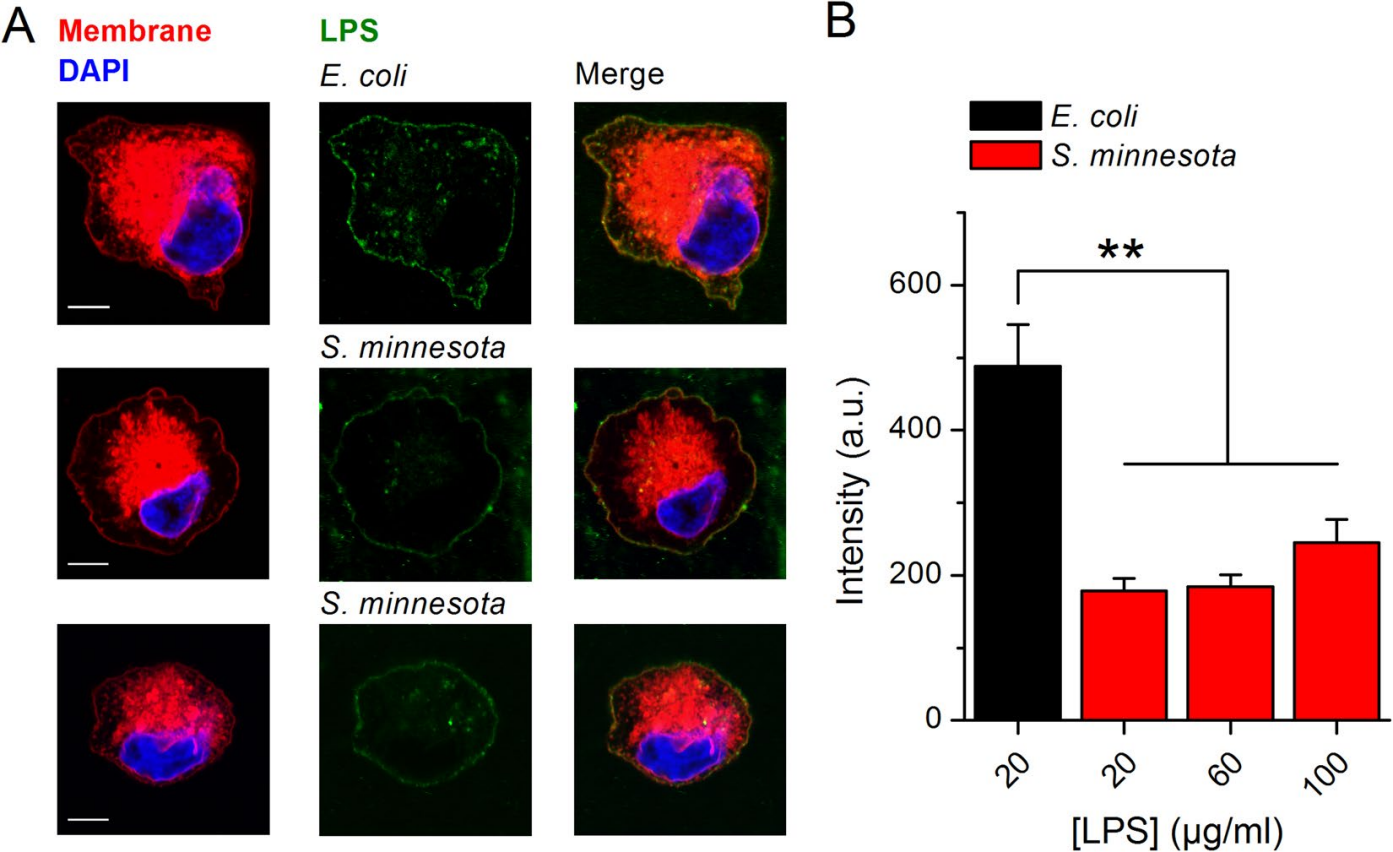
Spontaneous insertion of LPS from *E*. *coli* and *S*. *minnesota* into cellular membranes. (**A**) Representative Airyscan confocal images of CHO-TRPA1 cells stained with membrane mask (red), DAPI (blue) and treated with Alexa Fluor 488 LPS (green) from *E*. *coli* (20 µg/ml top panel) or *S*. *minnesota* (20 µg/ml and 100 µg/ml middle and bottom panel respectively). Scale bar, 5 µm (**B**) Quantification of Alexa Fluor 488 intensity present in the cellular membrane after treatment with LPS. Data is shown as mean ± s.e.m. from at least 10 cells. **P < 0.01, Mann-Whitney *U* test.

Since *S*. *minnesota* LPS does not activate TRPA1 we assessed if both LPSs are able to partition into the cellular membranes. Using confocal microscopy and fluorescent LPS from both *E*. *coli* and *S*. *minnesota* we found that both compounds insert into the cellular membranes of CHO-TRPA1 cells (Fig. 2A,B). Quantification of LPS present in the cellular membranes revealed lower intercalation of *S*. *minnesota* LPS in the bilayer (Fig. 2B). Increasing concentrations of *S*. *minnesota* LPS to 60 and 100 µg/ml (Fig. 2A,B) slightly enlarged insertion in the cellular membranes. Since both LPS insert in the plasma membrane, the lack of TRPA1 activation by LPS from *S*. *minnesota* seems to be also associated with a differential effect on properties of the membrane.

### Differential effects of LPS from *E*. *coli* and *S*. *minnesota* on membrane properties

As biological membranes are highly complex systems, artificial membranes are often utilized as tools for studying bilayer properties such as order/fluidity, phase transitions, as well as interactions between chemicals and membrane lipids. Giant unilamellar vesicles (GUVs) are one of the most commonly used model membrane systems, where lipid phase separation induced by changes in temperature or chemical insertion can be studying by measuring partitioning of fluorescent probes into different membrane phases.

To test the effects of LPS on lipid bilayers we first used 1,2-dipalmitoyl-*sn*-glycero-3-phosphocholine (DPPC), which forms stable and well-characterized unilamellar lipid vesicles. DPPC phase behavior displays distinctive lamellar phases: fluid phase occurs above 42 °C, the ripple phase around 37–38 °C and below that temperature the gel phase^19,20^. To record changes in the membrane properties we used fluorescent probe Laurdan (6-lauroyl,1-2-dimethylamino naphthalene), which displays a red-shifted emission when in contact with water molecules that penetrate the membrane upon increase in local disorder^21^.

**Figure 3:**
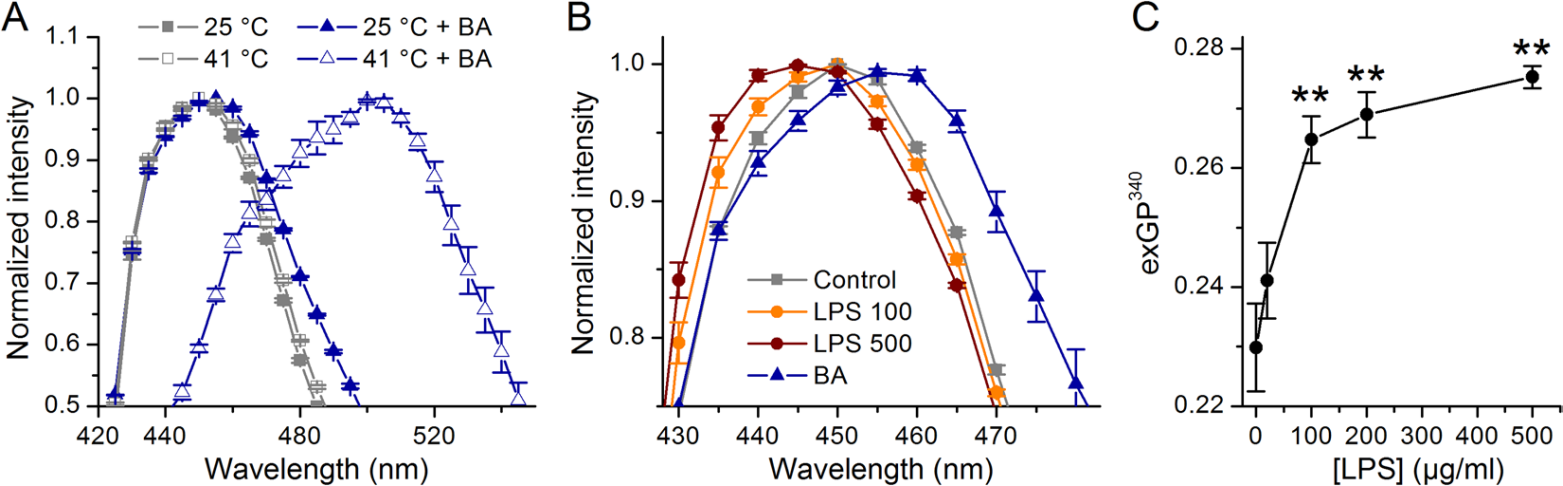
Effects of *E*. *coli* LPS on Laurdan’s emission spectrum in DPPC GUVs. (**A**) Laurdan spectra obtained at 25 °C and 41 °C in control and after treatment with BA (100 mM, 10 min). (**B**) Laurdan spectra recorded at 37 °C in control and after treatment with *E*. *coli* LPS (100 µg/ml or 500 µg/ml) or 100 mM of BA. (**C**) Laurdan exGP^340^ determined at 37 °C, as function of *E*. *coli* LPS concentration. Data from at least 3 samples performed at least in triplicate. **P < 0.01; Mann-Whitney *U* test.

Because membrane disorder is strongly correlated with the amount of water penetration into the bilayer, probe shifting towards the blue range (440 nm) indicates tight lipid packing (gel phase), whereas increased emission at 490 nm specifies disordered, fluid phase. Emission spectra of Laurdan solubilized in DPPC GUVs present typical curve with maximal emission at approximately 445–450 nm when measured at 25 °C (Fig. 3A). Heating from 25 °C to 41 °C elevated the right arm of the emission spectra, as expected from a fluidizing effect (Fig. 3A). At 25 °C, treatment with benzyl alcohol (BA, 100 mM), a known bilayer disordering agent^22,23^, also increased the right arm of the spectrum curve (Fig. 3A). Application of BA at 41 °C resulted in an emission maximum at approximately 500–505 nm, and the appearance of a “shoulder” at ~440 nm (Fig. 3A). Incubation of DPPC GUV with *E*. *coli* LPS shifted Laurdan’s emission spectrum in the blue direction, while the opposite was observed for BA (Fig. 3B).

To further describe the spectrum shifts we determined the generalized polarization (GP) parameter, as previously described^21,24^. In simple one-component membranes, GP values between −0.3 to 0.3 correspond to the fluid and between 0.5 and 0.6 to gel phase^24^. We found that the GP parameter determined at 340 nm (exGP^340^) decreased significantly from 0.230 ± 0.007 in control (37 °C) to −0.07 ± 0.04 in BA 100 mM, indicating membrane disordering. We also observed an exGP^340^ increase in a LPS concentration-dependent manner (Fig. 3C), indicating membrane ordering.

**Figure 4:**
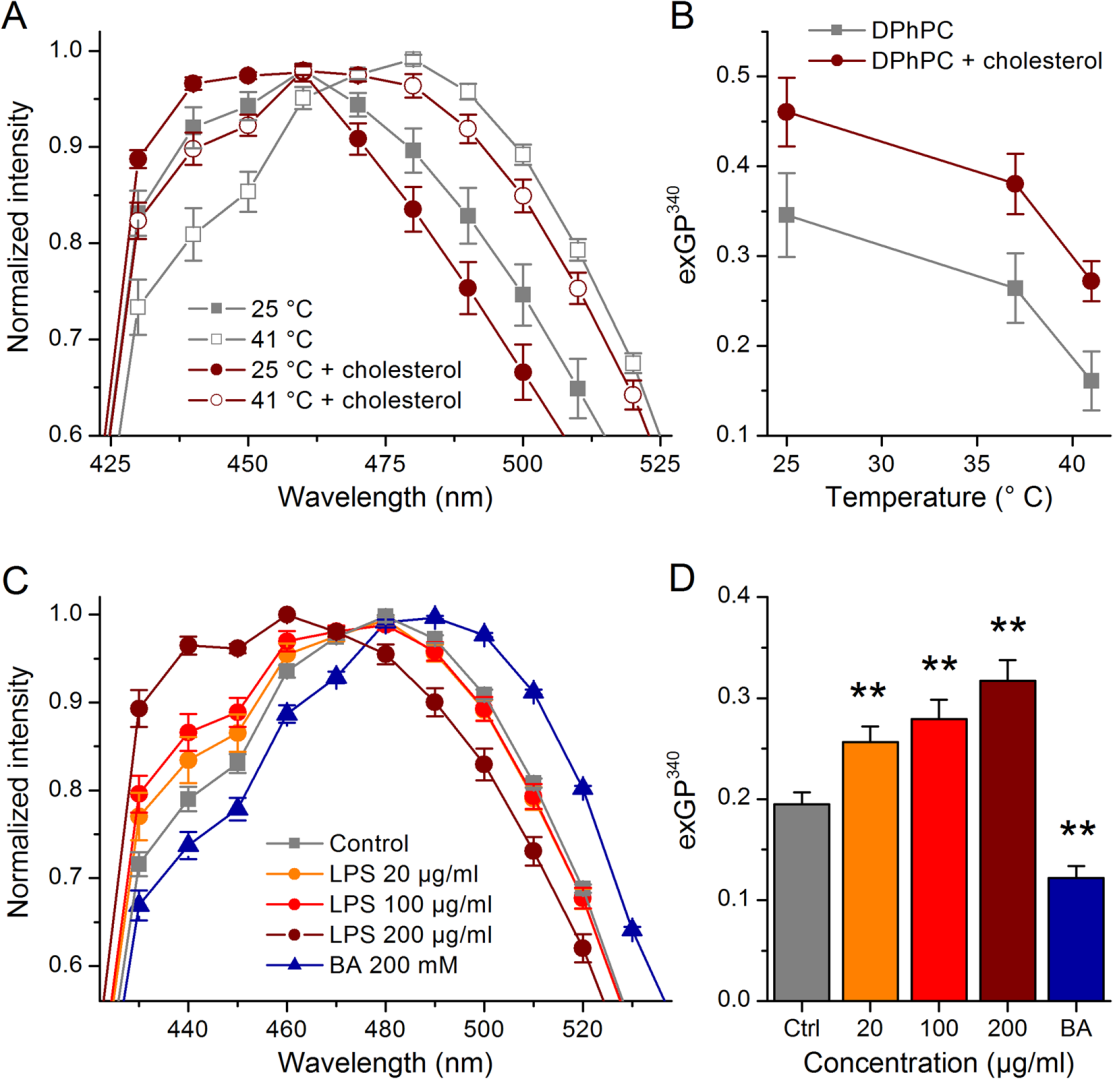
Effects of *E*. *coli* LPS on Laurdan’s emission spectrum in DPhPC GUVs. (**A**) Laurdan’s emission spectra obtained at 25 °C and 41 °C in control DPhPC GUVs and in DPhPC GUVs containing 20% cholesterol. (**B**) Laurdan exGP^340^ as function of temperature determined in DPhPC GUVs or DPhPC GUVs containing 20% cholesterol. (**C**) Effect of *E*. *coli* LPS (20, 100 and 200 µg/ml) or 200 mM BA on Laurdan’s emission spectrum recorded at 37 °C in DPhPC GUVs. (**D**) Laurdan exGP^340^ determined in control, in the presence of *E*. *coli* LPS (20, 100 and 200 µg/ml) or BA (200 mM). Data from at least three samples performed in at least triplicate. **P < 0.01; Mann-Whitney *U* test.

Next, we tested the effects of LPS in GUVs prepared with the DPPC derivative DPhPC, often used in the electrophysiological studies of protein-lipid interaction or ion channels activity, due to their high stability, low leakage, and lipid packing equivalent to physiologically relevant bilayers in the liquid-crystalline phase^25–27^. DPhPC has same length hydrocarbon chains of DPPC, but the addition of multiple methyl groups along acyl chains induces higher structural stability and lower lateral diffusion^27^. DPhPC has been shown to exhibit interesting membrane packing with the phosphocholine head group orientation being highly sensitive to temperature changes and solvent content^25–27^. When compared to DPPC GUVs, DPhPC vesicles displayed the shift into the disordered phase induced by temperature increase, with a spectrum peak at ~480 nm and the characteristic “shoulder” around 440 nm (Fig. 4A).

We tested whether we could detect ordering in the DPhPC GUVs by determining the effect of cholesterol, which modulates properties of the cellular membranes by influencing membrane thickness and stiffness and inducing formation of ordered domains^28,29^. At 25 °C, DPhPC GUVs containing 20% cholesterol displayed a Laurdan emission peak that was significantly shifted to lower wavelengths with respect to that obtained in pure DPhPC vesicles (Fig. 4A). Cholesterol also increased the spectrum “shoulder” at 440 nm. These effects were also observed when experiments were performed at 41 °C. Heating to 41 °C triggered fluidization of the DPhPC membranes, with a shift of the emission peak to 480 nm and a visible “shoulder” around 440 nm (Fig. 4A). Thus, the effects of heating alone mimic those of incubation with BA at high temperatures shown above in DPPC vesicles. This shows that DPhPC GUVs are more sensitive to membrane-perturbing factors when assessed through changes of Laurdan’s emission spectrum. The analysis of exGP^340^ changes clearly illustrated the disordering action of heating and the ordering effect of cholesterol (Fig. 4B). Application of *E*. *coli* LPS to DPhPC GUVs at 37 °C shifted Laurdan’s emission spectrum to lower wavelengths (Fig. 4C). As expected, BA shifted the emission curve towards the red region of the spectrum, with a decrease of emission at 440 nm and a displacement of the emission peak from 480 nm to 490 nm. The effects of *E*. *coli* LPS and BA were further validated by the analysis of exGP^340^ (Fig. 4D), demonstrating that LPS produces ordering and BA disordering in DPhPC GUVs.

**Figure 5:**
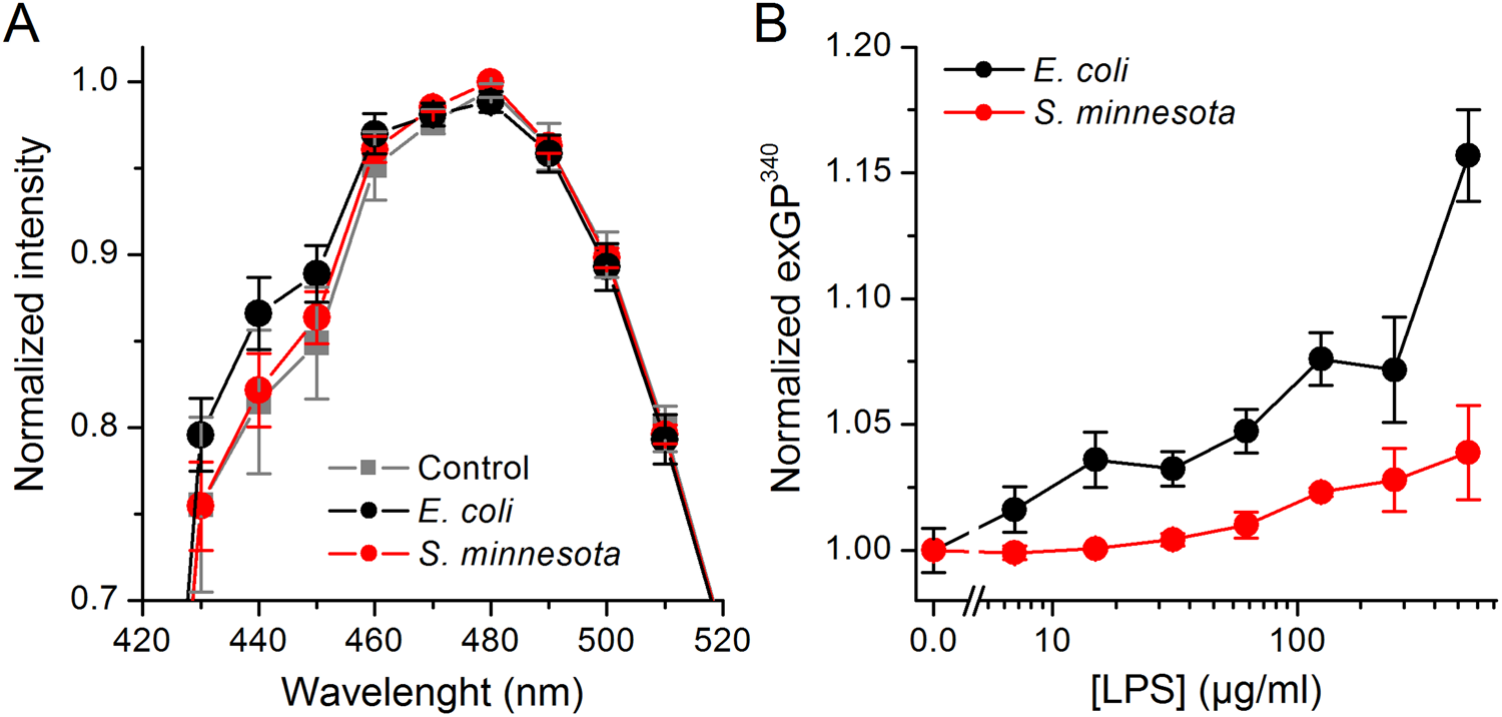
Differential effects of *E*. *coli* and *S*. *minnesota* LPS on Laurdan’s emission spectrum. (**A**) Laurdan’s emission spectra recorded at 37 °C in DPhPC GUVs in control and after treatment with 100 µg/ml of *E*. *coli* or *S*. *minnesota* LPS. (**B**) exGP^340^ determined at 37 °C in HEK293T cell exposed to *E*. *coli* or *S*. *minnesota* LPS applied at different concentrations. The values were normalized to those obtained in control condition. Data is shown as mean ± s.e.m. from at least three samples performed in at least triplicate.

We then performed experiments to compare the effects of *E*. *coli* LPS and *S*. *minnesota* LPS on Laurdan stained DPhPC GUVs. We found that 15 min incubation with the *S*. *minnesota* LPS (100 µg/ml) had no effect on Laurdan’s emission spectrum, whereas *E*. *coli* LPS shifted it towards the blue, with a visible increase at 440 nm (Fig. 5A).

We next tested whether our findings in GUVs could be corroborated in natural cell membranes. Treatment of HEK293T cells with *E*. *coli* LPS for 10 min increased Laurdan exGP^340^ in a concentration-dependent manner (Fig. 5B). In contrast, *S*. *minnesota* LPS induced significant changes in exGP^340^ only above 100 µg/ml, and at 500 µg/ml the effects were smaller than those produced by *E*. *coli* LPS. As expected, normalized exGP^340^ decreased from 1.000 ± 0.007 in control to 0.838 ± 0.026 in 125 mM BA.

**Figure 6:**
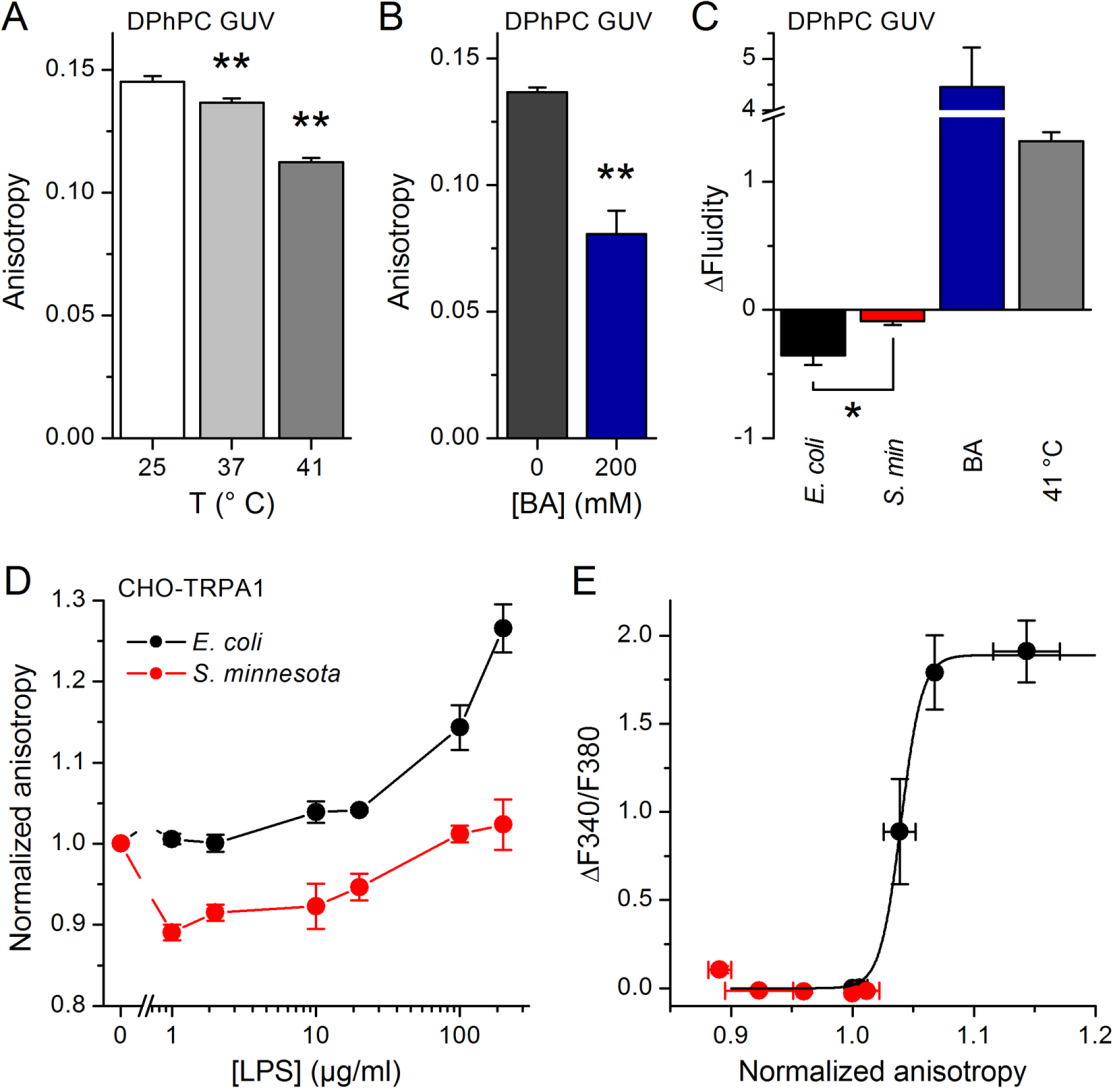
Differential effects of *E*. *coli* and *S*. *minnesota* LPS on the anisotropy of DPH fluorescence. (**A**) DPH fluorescence anisotropy determined at 25, 37 or 41 °C in DPhPC GUVs. (**B**) DPH fluorescence anisotropy obtained at 37 °C in DPhPC GUVs in control and in the presence of 200 mM BA. (**C**) Average change in membrane fluidity induced by *E*. *coli* LPS, *S*. *minnesota* LPS, 200 mM BA or heating to 41 °C. (**D**) Normalized anisotropy of DPH fluorescence determined at 37 °C in CHO-mTRPA1 cells treated with *E*. *coli* or *S*. *minnesota* LPS at different concentrations. The data is shown as mean ± s.e.m. from at least three experiments performed in triplicate. *P < 0.05; **P < 0.01, Mann-Whitney *U* test. (**E**) Relation between LPS-induced increased intracellular Ca^2+^ levels caused by TRPA1 activation and change in DPH fluorescence anisotropy recorded in CHO-TRPA1 cells upon application of *E*. *coli* or *S*. *minnesota* LPS (0–100 µg/ml). The black line represents the fit of the data for *E*. *coli* with a Boltzmann function of the form ΔF340/F380 = ΔF340/F380_Max_/(1 + exp((r − r_0_)/s_r_)), where ΔF340/F380_Max_ is the maximal Fura2 ratio, *r* is the normalized anisotropy, *r*_*o*_ is the value of normalized anisotropy for which the Fura2 ratio is half-maximal and *s*_*r*_ is the slope factor.

Next, we evaluated the effects of LPS on membrane properties using diphenylhexatriene (DPH), a compound whose fluorescence anisotropy is reduced upon increases in membrane fluidity^30,31^. Both heating and treatment with BA (200 mM) at 37 °C reduced DPH fluorescence anisotropy in DPhPC GUVs (Fig. 6A,B), which translated to an increase in membrane fluidity (Fig. 6C). Treatment with LPS from *E*. *coli*, but not from *S*. *minnesota*, significantly decreased fluidity (Fig. 6C). Anisotropy recordings in CHO-TRPA1 cells revealed that treatment with *E*. *coli* LPS produce lipid ordering (Fig. 6D,E). In contrast, *S*. *minnesota* LPS did not induce consistent changes. Similar results were obtained in HEK293T cells (data not shown).

**Figure 7:**
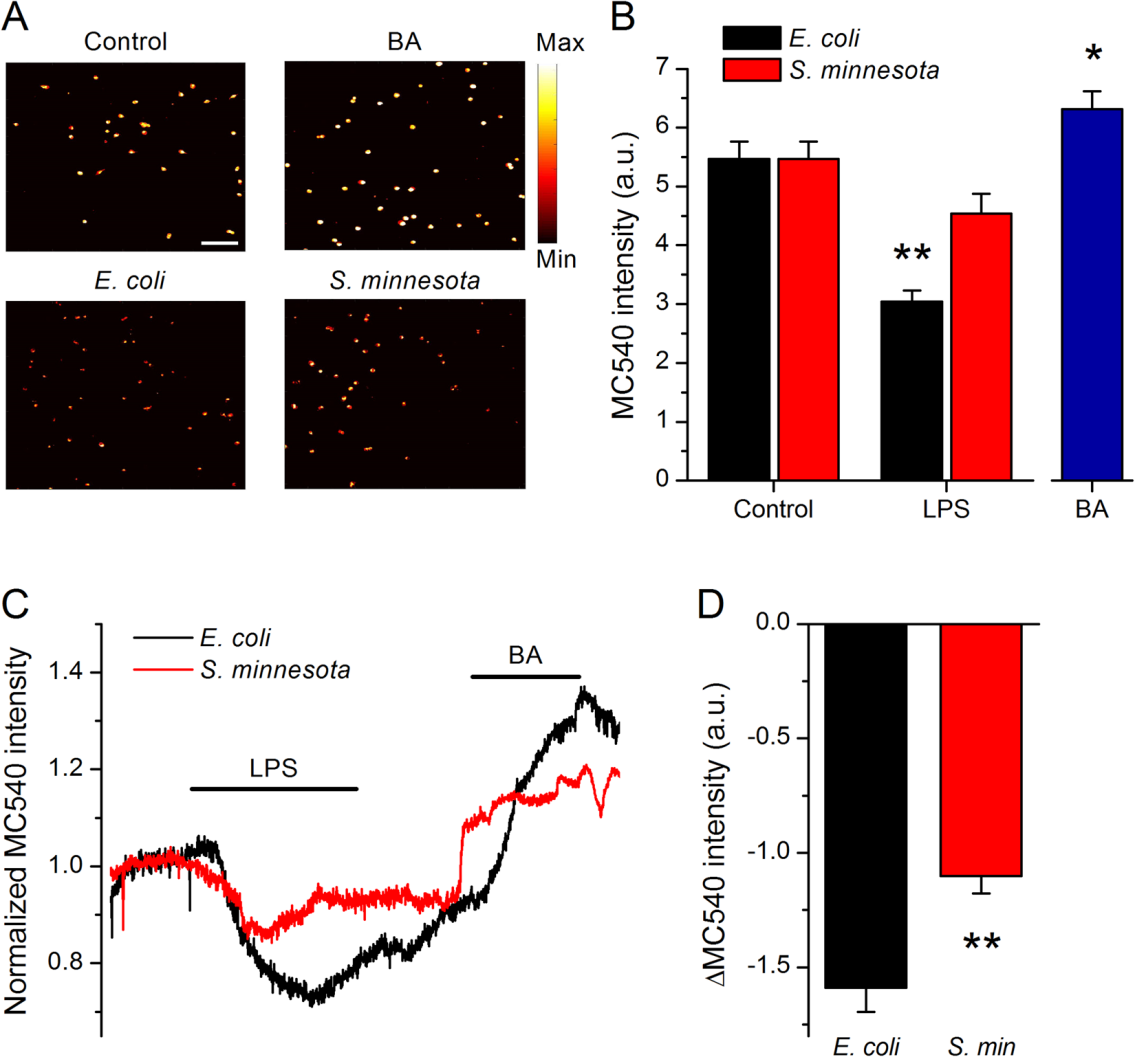
Changes in membrane MC540 fluorescence induced by LPS treatment. (**A**) Representative microscopic pseudo-colored images of HEK293T cells stained with MC540 (left, top panel) and pre-treated for 15 min with 125 mM BA (right, top panel) or 8 µg/ml *E*. *coli* LPS or *S*. *minnesota* LPS (bottom panels). Scale bar, 150 µm. (**B**) Average MC540 fluorescence intensity values obtained from determinations in the control, LPS or BA (n > 300). (**C**) Representative traces of MC540 fluorescence showing the effects of application of LPS from *E*. *coli* or *S*. *minnesota*. In both experiments, 125 mM BA was applied after LPS wash out. The horizontal lines indicate the duration of compound applications. (**D**) Average maximal changes in MC540 fluorescence intensity induced by *E*. *coli* (n = 40) and *S*. *minnesota* (n = 37) LPS. All recordings were conducted at 21 °C. *P < 0.05; **P < 0.01, Mann-Whitney *U* test.

To monitor variations in membrane viscosity we used merocyanine 540 (MC540), a lipophilic dye whose fluorescence increases upon binding to membranes with loosely packed, fluid-phase lipids, thus allowing monitoring membrane viscosity^32,33^. BA treatment enhanced MC540 fluorescence intensity (Fig. 7A,B), whereas *E*. *coli* LPS decreased MC540 fluorescence intensity, and *S*. *minnesota* LPS had a minor, though visible reducing effect (Fig. 7A,B). Finally, we evaluated the dynamic changes of MC540 fluorescence intensity upon LPS application. Cells perfused with MC540 display a steady fluorescence intensity, which was followed by a significant reduction upon addition of *E*. *coli* LPS (Fig. 7C,D). *S*. *minnesota* LPS caused much less reduction of MC540 intensity, indicating a weaker ordering effect. A subsequent application of BA increased MC540 fluorescence, demonstrating its expected fluidizing action (Fig. 7C).

## Discussion

Bacterial LPS, an outer wall glycolipid released upon cellular lysis and replication was recently unveiled as a natural activator of the chemosensory cation channel TRPA1. Although it has been established that TRPA1 activation by LPS is independent of the canonical receptor TLR4^6^, the exact underlying mechanism remains unknown. Using three fluorescent membrane dyes, we here demonstrate that *E*. *coli* LPS induces significantly stronger mechanical alterations in the plasma membrane than *S*. *minnesota* LPS, and that this relates to the ability of these endotoxins to activate TRPA1.

It must be noticed that the *E*. *coli* LPS concentrations required to activate TRPA1 are about 2 orders of magnitude higher than those typically used experimentally to activate the canonical TLR4 pathway *in vitro*. However, it has been reported that pathological conditions such as severe endotoxemia can induce rise of plasma LPS levels into the µg/ml range^34^, which corresponds to the range needed for TRPA1 channel activation. These relatively high LPS concentrations are also present in urine and locally inflamed tissues^35^ and are typically used in current experimental models of endotoxin challenge in LPS-evoked pain behavior experiments in animals^36^ and LPS-induced airway inflammation^37–39^. It is indeed conceivable that LPS concentrations can locally rise significantly during acute infection due to accumulation of bacterial debris in the very small volumes of acute dental abscesses and micrometer-thin mucosal layers at epithelial barriers. Thus, our findings are likely to be relevant for certain acute infection scenarios, as well as for the interpretation of current experimental models of endotoxin challenge.

Our findings provide conclusive support to previous reports on ordering of lipid membranes by *E*. *coli* LPS^14,40–43^. This endotoxin was shown to rapidly integrate into 1,2-dimyristoyl-*sn*-glycero-3-phosphocholine (DMPC) membranes, inducing significant reduction of the elastic strength, resulting in vesicles size reduction^44^. Correspondingly, *E*. *coli* LPS produced volume decrease, increased membrane viscosity, as well as reduction of stress tolerance in red blood cells^41–43^. Likewise, *E*. *coli* LPS application to hepatocytes caused increase in viscosity and delay in the membrane fluidization induced by heating^14^.

Our results shed new light on the mechanism underlying activation of TRP channels by LPS. First, they provide support to the hypothesis of mechanical perturbations as being the trigger for channel activation, since the effects of *E*. *coli* LPS on membrane order, determined with Laurdan, DPH and MC540, occurred in a concentration range overlapping with that required for TRPA1 activation. Second, they provide hints on how *E*. *coli* LPS activates TRPA1. The relationship between the amplitude of the Ca^2+^ responses and the increase in DPH fluorescence anisotropy induced by *E*. *coli* LPS in CHO-TRPA1 cells has a sigmoidal shape, displaying a dynamic range around the 4% increase in anisotropy and a saturation phase beyond ~9% increase in anisotropy (Fig. 6E). These values suggest that TRPA1 is very sensitive to the sort of membrane mechanical alterations produced by LPS that are detected by DPH (i.e., decreased fluidity). Further insight is provided by the result that *S*. *minnesota* LPS decreased the fluorescence intensity of MC540. This, together with our membrane staining results (Fig. 2), demonstrates that *S*. *minnesota* LPS does interact with the plasma membrane lipids. However, this LPS was ineffective on TRPA1 and on the fluorescence properties of Laurdan and DPH. Such results, together with the fact that both Laurdan and DPH report changes in membrane order at positions below the lipid heads, suggest that the structures that render TRPA1 sensitive to *E*. *coli* LPS are distal to the extracellular phase, within the lipid bilayer.

The recently elucidated structure of TRPA1^45^ suggests a large contact surface with membrane lipids. Therefore, changes in membrane physical properties such as order, tension, thickness and curvature resulting from the insertion of LPS in the close vicinity of the channel might in principle alter the TRPA1-bilayer interaction that may induce channel opening^46^. However, if considering that bilayer stretching is associated with membrane fluidization^47^, our results indicate that LPS produces lateral compression. This indicates, in turn, that the mechanism underlying TRPA1 activation by LPS is different from that operating in “classical” mechanosensitive channels activated by membrane stretch^48–50^.

The structural model of TRPA1 also reveals the presence of 14–18 ankyrin repeats in the N-terminus of the protein. These may function as elastic anchors to cytoskeletal proteins and thereby regulate the movement of channel gates upon LPS-induced mechanical alterations in the plasma membrane. The N-terminus also contains several cysteine and lysine amino acid residues that were reported to be required for covalent binding of electrophilic agonists^51,52^. It could be therefore envisaged that LPS activates TRPA1 through the generation of an endogenous electrophilic compound. However, it must be noted that TRPA1 activation by LPS was preserved in cell-free inside-out patch-clamp recordings, which discards an critical role of intracellular soluble mediators and interaction partners in this mechanism^6^.

A remaining question is why *S*. *minnesota* LPS has weaker effects in the plasma membrane mechanical properties than *E*. *coli* LPS? Our experiments with fluorescence-labeled LPS indicate that one of the reasons underlying this difference may be the lower efficiency of insertion of *S*. *minnesota* LPS in the plasma membrane. It is likely that the different effects of these molecules obey to the distinct structures of their hydrophobic lipid A moieties. *E*. *coli* LPS has 6 acyl chains that are thought to adopt a conical shape upon insertion, whereas *S*. *minnesota* LPS has 7 acyl chains and a cylindrical shape. It has been shown that insertion of LPS alters the shape of lipid vesicles, causing concentration dependent shape fluctuations such as bead-like protrusions^53^. We speculate that conically-shaped lipid A moieties cause more alterations in the membrane structure than cylindrical ones. This is supported by the strong correlation we previously reported between the lipid A shape and the ability of different LPS molecules to activate TRPA1 *in vitro* and to cause inflammation *in vivo*^6^. Of note, our findings are consistent with previous studies reporting, for example, weaker effects of *S*. *minnesota* lipid A respect to *E*. *coli* lipid A on TNF-α induction and NF-κB activation in human-derived cells^54^. Thus, although surprising, it seems clear that relatively small modifications of the lipid A structure may have a strong impact on the functional properties of LPS. The elucidation of the precise molecular mechanisms underlying the distinct influence of hexa- and hepta-acylated lipid A moieties on the mechanical properties of plasma membranes is a fascinating issue, but it is beyond the scope of the present study.

Taken together, our findings strongly support the hypotheses that TRPA1 activation by LPS results from mechanical perturbations in the plasma membrane, and that TRP channel-mediated chemosensation may result, not only from classical compound-receptor interactions, but also from primary mechanosensory mechanisms^55,56^. Our results represent a significant advance in the understanding of newly-described mechanisms of innate defense mechanisms against bacterial infections, whereby activation of TRP channels trigger protective responses in sensory neurons and are therefore at the center of a novel field of research on neuro-immune interactions^57^.

## Methods

### Cell culture

Human embryonic kidney (HEK293T) cells from the European Collection of Cell Culture (Salisbury, UK) were grown in Dulbecco’s modified Eagle’s medium (DMEM) containing 10% (v/v) fetal calf serum, 2 mM L-glutamine, 2 units/ml penicillin and 2 mg/ml streptomycin (Gibco/Invitrogen, Carlsbad, CA, USA) at 37 °C in a humidity-controlled incubator with 10% CO_2_. Chinese hamster ovary (CHO) cells from the American Type Culture Collection were grown in DMEM containing 10% fetal bovine serum, 2% glutamax (Gibco/Invitrogen), 1% non-essential amino acids (Invitrogen) and 200 µg/ml penicillin/streptomycin at 37 °C in a humidity controlled incubator with 5% CO_2_. As TRPA1 expression system we used tetracycline-inducible CHO cells stably transfected with mouse TRPA1 (CHO-TRPA1).

### Intracellular Ca^2+^ imaging experiments

Experiments were performed using a variant of previously published method^58^. Briefly, CHO-TRPA1 cells were plated at a density 20000 cells/well onto poly-D-lysine-coated flat bottom 96-well microtiter plates (Greiner Bio-One, Belgium) and left to attach overnight. For experiments cells were incubated with 2 µM Fura-2 AM (Biotium, Hayward, CA, USA), at 37 °C for 60 min. Loaded cells were washed, re-suspended in the assay buffer (HBSS, 2 mM CaCl_2_, 2.5 mM probenecid, 10 mM HEPES adjusted with NaOH to pH 7.4) and placed into the FlexStation 3 (Molecular Devices, Sunnyvale, USA) to monitor fluorescence before and after the addition of the compounds of interest. The intracellular Ca^2+^ responses were determined at 37 °C from fluorescence emissions at 510 nm elicited by alternating excitation at 340 and 380 nm.

### Confocal microscopy

CHO-TRPA1 cells were seeded in glass coverslips and exposed to LPS from *E*. *coli* Alexa Fluor™ 488 Conjugate or *S*. *minnesota* Alexa Fluor™ 488 Conjugate (ThermoFisher Scientific, Waltham, MA, USA) during 10 min and CellMask™ Deep Red Plasma Membrane Stain (ThermoFisher Scientific) during 2 min. After treatment, cells were washed and fixed with cold paraformaldehyde and mounted in glass slides using DAPI-containing mounting solution (VectaShield, Vector Laboratories, Burlingame, CA, USA). The confocal images of labeled cells were collected using the optimal pinhole size for the 63X oil objective of a Zeiss LSM 880 Airyscan microscope (Carl Zeiss AG, Oberkochen, Germany). For quantification of cell images we analyzed a minimum of 10 cells from different slides per condition using the ImageJ software^59^.

### Preparation of giant unilamellar vesicles (GUVs)

1,2-dipalmitoyl-*sn*-glycero-3-phosphocholine (DPPC, 25 mg/ml) or 1,2 Diphytanoyl-*sn*-Glycero-3-Phosphocholine (DPhPC, 25 mg/ml) from Avanti Polar Lipids, Inc. (Alabaster, AL, USA) were dissolved in chloroform (Sigma-Aldrich, Bornem, Belgium) to obtain 20 mM stock solution, which were further diluted with chloroform to final working concentration of 10 mM. The stock of cholesterol (1 mM) (Sigma-Aldrich) was also prepared in chloroform. We produced GUVs with incorporated fluorescent probes (Laurdan and DPH) using a 1/800 probe-to-lipid ratio. GUVs were prepared by the electroformation method (hydration of a dry lipid film in an oscillating electric field) using a Nanion Vesicle Prep Pro setup (Nanion Technologies GmbH, Munich, Germany). Lipid solutions (40 µl) containing 10 mM DPPC or DPhPC, with or without fluorescent probes, or 10 mM DPhPCs with 20% cholesterol (with or without fluorescent probes) were deposited on the conductive side of indium tin oxide (ITO) coated glass electrode and evaporated under vacuum to remove chloroform traces. After total solvent evaporation, a greased O-ring was placed around the dried lipid film and filled with 1 M sorbitol solution, pH 7.0. Then, a second ITO-electrode was placed on the ring. GUV formation was induced by application of an alternating voltage of 3 V peak-to-peak for 2 h with a frequency of 5 Hz, while keeping the temperature at 60 °C (DPPC GUV) or 37 °C (DPhPC GUV). Formation of GUV was followed by visualization with an upright bright field microscope (amplification 40X). Last, GUVs were plated into flat-bottom 96-well microtiter plates (Greiner Bio-One), and treated with LPS extracted from *E*. *coli* (serotype 0127:B8, Sigma-Aldrich) or *S*. *minnesota* (Sigma-Aldrich) or the fluidizing agent benzyl alcohol (Sigma-Aldrich).

### Fluorescent measurements using Laurdan, DPH or MC540

A 0.8 mM stock solution of Laurdan (6-Dodecanoyl-N,N-dimethyl-2-naphthylamine) was prepared in methanol or chloroform (Sigma-Aldrich). Next, 1.5 µM Laurdan was prepared in phosphate buffered saline (PBS) to stain HEK293T or CHO-TRPA1 cells (1 × 10^6^ cells/ml) for 30 min at 37 °C. After incubation, cells were washed and suspensions of 100 μl were aliquoted into flat-bottom 96-well microtiter plates (Greiner Bio-One). Steady-state Laurdan fluorescence measurements were performed using a FlexStation 3 Benchtop Multi-Mode Microplate Reader and the SoftMax Pro Microplate Data Acquisition & Analysis Software (Molecular Devices). Laurdan excitation spectra were obtained in the range of 300–420 nm, using both 435 and 490 nm emission wavelengths. Laurdan emission spectra were recorded in the range from 420–550 nm, using both 340 and 410 nm excitation wavelengths. Readings were made at 37 °C for all the samples or between 25–41 °C when characterizing the effects of temperature. Generalized polarization (GP) values were calculated using emission values at 440 nm and 490 nm, and excitation at 340 nm, according to the formula^21^

$${{\rm{exGP}}}^{340}=({{\rm{I}}}_{440}-{{\rm{I}}}_{490})/({{\rm{I}}}_{440}+{{\rm{I}}}_{490}).$$

A 1 mM stock solution of 1,6-diphenyl-1,3,5-hexatriene (DPH, Sigma-Aldrich) was prepared in dimethyl sulfoxide (DMSO; Sigma-Aldrich) and working solution of 10 µM was prepared in PBS. Cells were stained as described above for Laurdan. DPH anisotropy (r) was monitored with an excitation wavelength of 365 nm and emission wavelength of 430 nm using a Flexstation 3 Benchtop Multi-Mode Microplate Reader and the SoftMax Pro Microplate Data Acquisition & Analysis Software (Molecular Devices). A linearly-polarized excitation beam was generated by a vertical polarizer that excites DPH with transition moments aligned parallel to the incident polarization vector. The resulting fluorescence intensities in both parallel (I_VV_) and perpendicular (I_VH_) directions to that of the excitation beam were recorded and the fluorescence anisotropy can be calculated by: r = (I_VV_ − G∙I_VH_)/(I_VV_ + 2 G∙I_VH_), where G = I_HV_/I_HH_. The relation between anisotropy (r) and polarization (P) was determined as P = 3r/(2 + r) and the membrane fluidity was calculated as the inverse of fluorescence polarization (1/P)^60^.

A stock solution of merocyanine 540 (MC540; 54 mM; Sigma-Aldrich) was prepared in DMSO. From this 2.7 µM MC540 solutions were prepared in Krebs buffer (containing in mM: 150 NaCl, 6 KCl, 1 MgCl_2_, 1.5 CaCl_2_, 10 HEPES, 10 glucose and adjusted with NaOH to pH 7.4). HEK293T cells (2 × 10^4^ cells/ml) were seeded on 18 mm glass coverslips with thickness of 0.13–0.16 mm (Gerhard Menzel GmbH, Germany) coated with poly-L-lysine (0.1 mg/ml) (Sigma-Aldrich). These cells were grown in DMEM/F12 medium at 37 °C in a humidity-controlled incubator with 10% CO_2_ for minimum 2 h. After incubation, cells were washed twice with Krebs, re-suspended in the 2.7 µM MC540 solution and incubated in the dark for 15 min at 37 °C. After incubation, cells were re-suspended in Krebs solutions containing LPS (0.01 to 500 µg/ml) or control Krebs solution with or without 125 mM benzyl alcohol and incubated for 15 min at 37 °C. Changes in membrane fluidity between different treatment groups were detected with an Olympus BX51W1 inverted fluorescence microscope equipped with an UMPLANFL 10X objective. Extracellular perfusion was applied with a Warner Instruments CL-100 multichannel system. Cells were excited using a Polychrome V light source and images acquired with an Andor iXon 888 camera coupled with the TILL Photonics Live Acquisition 2.3.0.18 software. Fluorescence from HEK293T cells was recorded at emission wavelength 560–570 nm and excitation wavelength of 488 nm. For quantification of cell images we analyzed a minimum of 300 cells from 4 different slides per condition using the ImageJ software^59^.

### Data and statistical analysis

Data are presented as mean ± s.e.m. using Origin 9.0 (OriginLab Corporation). We used the non-parametric Mann-Whitney test to assess statistical significance with the GraphPad Prism software. In the Figures, the asterisks indicate the level of statistical significant difference (*P < 0.05 and **P < 0.01).
